# Tumors

**Published:** 1890-01

**Authors:** A. M. Potter


### Tumors.

BY A. M. POTTER, D.D.S.

This is a broad subject and for anything like a complete discussion, would greatly exceed in length the possibilities of the present short paper.

The study of tumors is to the practitioner of dentistry an all important one, and to the man who may extend his work into the domain of oral surgery, it is one which, from its frequency, its mortality and from its very uncertainty, one of all absorbing interest.

In the first case its chief importance lies in the ability to recognize and diagnose a malignant growth, which seen in its earlier stages may, by its complete removal, give immunity to the patient, which had it progressed to a stage when the surrounding structures were involved, the patient would be beyond all operative and remedial measures.

I wish to emphasize—not the particular value of knowledge to operate, by the dental practitioner—but the knowledge to appreciate the condition, to be able to so diagnose and advise, that relief may be sought immediately. There are undoubtedly many cases where this help would be, and is, the only necessary help to, perhaps, save a patient's life.

This may be considered peculiarly the office of the dentist, as in our practice we many a time have an opportunity to see these growths long before a surgeon would be consulted.

It is found, after a careful consultation of the reports of eminent pathologists, that once again as many malignant growths begin upon the tongue, lips, or cheeks, as upon any other portion of the body; and so from the reasons just given, this paper will be confined chiefly to the diagnosis or points of difference between benign and malignant tumors.

For many years previous to the introduction of the microscope, classifications of tumors were attempted, and since its introduction the classification is more confused than ever. The fact is, the microscope presents actual appearances on its field, but as there are few expert microscopists and the specimens are taken at different stages of development, they may vary greatly, according to position or circumstances.

The elaborate and scholarly classifications of Bilroth, Virchow, Paget, McKenzie, Holmes, Brodie, and others indicate the profound study which is given this subject, but from the very fact of so many classifications, it has a tendency to bewilder the student, owing to the different nomenclature used by different authors, thus: the "Myeloma" of Sir James Paget, is the "giant celled sarcoma" of Virchow; the "recurrent fibroid" is the "spindle shaped sarcoma" of the German pathologist; each prominent pathologist having a different classification.

As a means of diagnosis between benign and malignant tumors, I mean, of course, primary diagnosis, the microscope is of little practical use, and to quote the language of Dr. Savage: "The question of malignancy is not to be determined histologically, the experienced surgeon decides without much reference to histology and is generally right, where the pure histologist is generally wrong."

The definition of a tumor, according to Paget, is: "A new growth which is an addition to the normal structures of the body with appearance of inherent power, irrespective of the maintenance of the rest of the body, discordant with the normal type and with no seeming purpose."

The first formation that is noticed in the growth of a tumor, is a minute mass of protoplasm, the product of the connective tissue corpuscle. At this period of development it is impossible to tell whether it is to be innocent or malignant, and if, as the tumor grows, it takes upon itself the nature of fat, fibre, flesh, bone, or any normal adult tissue of the body, the diagnosis is that it is innocent. If, on the other hand, a proliferation of similar cells takes place and do not proceed to a complete formation, if they are irregular masses of tissue largely supplied with blood vessels, then the tumor will assume one of the many forms of corcinoma and it can safely be added that all tumors that can not be proved benign should be considered malignant and treated as such to secure the greatest good to the patient.

In volume they range from the size of a small shot to a volume greater than that of a patient's body.

The color varies with the amount of blood vessels.

Nævus is purple; fatty tumors, yellow; fibrous, whitish; &c., according to the nature of the tissues involved.

Classifying tumors clinically, we may say that there are two great typical divisions—innocent and malignant—with an intermediate variety which may partake of the nature of each, with of course an understanding that one division may overlap and merge into another.

Innocent tumors may be distinguished by the following characteristics:

1. Harmless in reference to surrounding structures.
2. With exception of recurrent fibroids they are not liable to return after proper extirpation.
3. In texture they resemble some one of the normal adult tissues of the body.
4. As a rule they are unattended by any marked constitutional disturbances.

The following diagnostic signs characterize malignant growths:

1. In microscopic structure they differ entirely from normal tissues of the body.
2. They are disposed to soften and ulcerate.
3. They do not enlarge continuously, but become irregular and lobulated with offshoots; innocent tumors, on the contrary, are generally round and grow in one volume.
4. They are marked by persistent hemorrhage.
5. The fetor is easily recognizable.
6. They have a tendency to invade surrounding structures, to produce secondary deposits, and to invade distant organs.
7. They are liable to return after extirpation.
8. Produce constitutional disturbances.
9. Malignant growths attack most frequently the glandular organs, while benign tumors infect the skin, cellular-adipose tissue, etc., etc.

There is another class of tumors which may partake of the nature of either of the above; it is what is known as the sarcomatous tumor; by this is understood a matrix or stroma, surrounding cells of various character, the precise character of the cell element giving the character to the formation, hence the great variety of names.

Greatest benignancy and greatest malignancy may be united in the sarcomatous group; and, says Billroth, the prominent surgeon and pathologist, "I can assure you that sarcomata of the most similar histological qualities may differ entirely in course."

There is one or two forms of epulis that is frequently met by the dentist; that known as the fungoid epulis, springing from the pulp cavity of a badly decayed tooth, and the fibrous epulis, which springs from the alveoli and is connected distinctly to the periosteum; it is generally peduncled and can be handled without pain; absence of disease in adjacent glands and of tendency to infiltration, diagnose it from malignant formations. As a rule time is lost prescribing medicines for these tumors; the knife is the only resort.

To sum up in a few words, the nearer a tumor approaches in its structure the perfectly developed formations of the human body, the more likely it is to be innocent; and the greater the departure from this standard, or in other words the nearer the resemblance to imperfectly formed or abnormal structure, the more certain is the growth to be malignant.

